# Altered cortical activation and functional connectivity during fixed phrase verbal fluency in children with autism spectrum disorder

**DOI:** 10.3389/fpsyt.2026.1786619

**Published:** 2026-08-14

**Authors:** Bin Yu, Hong-Xia Li, Xi-Ning He, Yun-Sheng Fu, Xiao-Hong Zhang, Ge Lei, Yu Zhao, Cheng-Feng Zhao, Xiu-E Shi, Jian-Min Lv

**Affiliations:** 1 Pediatric Medical Rehabilitation Center, Shaanxi Provincial Rehabilitation Hospital, Xi’an, Shaanxi, China; 2 Rehabilitation Science Institute, Shaanxi Provincial Rehabilitation Hospital, Xi’an, Shaanxi, China

**Keywords:** autism spectrum disorder, cortical activation, executive function, functional connectivity, functional near-infrared spectroscopy, intellectual disability, verbal fluency task

## Abstract

**Background:**

This study examined prefrontal activation and functional connectivity during a fixed phrase verbal fluency task (VFT) in children with Autism Spectrum Disorder (ASD) compared to basic information- and Full-Scale Intelligence Quotient (FSIQ)-matched children with intellectual disability (ID), to identify neural mechanisms underlying executive dysfunction specific to ASD beyond general cognitive impairment.

**Methods:**

The sample included 33 children diagnosed with ASD and 21 with ID, all with FSIQ < 70. Cortical hemodynamics were recorded using a 19-channel functional near-infrared spectroscopy system during a standardized phonemic VFT. Analysis focused on changes in oxyhemoglobin (Oxy-Hb) concentrations within prefrontal cortex (PFC) regions.

**Results:**

Compared to the ID group, children with ASD showed significantly reduced cortical activation in prefrontal regions, including Channels 1 (t = -4.272, *p* < 0.01) and 2 (t = -3.827, *p* < 0.01), corresponding anatomically to the right inferior frontal gyrus. Functional connectivity was also significantly lower in the ASD group (*p* < 0.05), with a mean connectivity value of 0.191 (SD = 0.130) versus 0.230 (SD = 0.155) in the ID group. Furthermore, Oxy-Hb changes in prefrontal Channels 1 (r = -0.852, *p* < 0.001) and 2 (r = -0.606, *p* < 0.001) were negatively correlated with Childhood Autism Rating Scale total scores, indicating that decreased prefrontal activation was associated with increased clinical symptom severity.

**Conclusions:**

These findings demonstrate distinct alterations in cortical activation and functional connectivity during VFTs in children with ASD. These findings suggest that disrupted coordination among brain regions contributes to impairments in phonemic fluency and executive control in ASD. Children with ASD exhibited reduced PFC activation accompanied by weakened functional connectivity during the VFT.

## Introduction

Autism Spectrum Disorder (ASD) is a neurodevelopmental condition characterized by persistent impairments in social communication and interaction, alongside restricted and repetitive patterns of behavior or interests (1, 2). Epidemiological data indicate a steadily increasing prevalence worldwide, with recent estimates indicating that approximately 1 in 36 individuals is affected (3). This rising prevalence underscores the growing public health importance of ASD, as well as its considerable socioeconomic impacts on affected individuals, their families, and healthcare systems. Consequently, advanced research into the neurobiological mechanisms underlying ASD is of critical importance to inform the development of targeted, evidence-based interventions aimed at improving clinical outcomes.

Clinically, children with ASD often exhibit pronounced impairments that exceed what would be expected based on their cognitive abilities, even when compared to children with intellectual disability (ID) matched for similar intellectual functioning, as measured by Full-Scale Intelligence Quotient (FSIQ) below 70 (4). Social adaptation in ASD reflects the complex interaction between core symptomatology, such as difficulties in social communication challenges, and individual cognitive strengths and weaknesses. Cognitive functioning, encompassing general intellectual ability and specific domains such as executive function (EF), plays a crucial role in how individuals manage social demands (5, 6). Given the fundamental role of EF in cognitive and adaptive functioning, interventions targeting this domain hold promise for substantially improving social outcomes in ASD (7). However, the neurocognitive mechanisms underlying executive dysfunction in ASD remain poorly understood (8).

EF refers to a set of higher-order cognitive control processes, including working memory, cognitive flexibility, and inhibitory control, that enable the regulation of goal-directed behavior and adaptive responses. Deficits in EF have been robustly associated with impairments in planning, self-regulation, and social functioning, particularly within neurodevelopmental research (9, 10). The verbal fluency task (VFT) is widely used as an experimental paradigm for assessing phonemic fluency and executive control under time pressure, as it requires the rapid generation of words according to specific semantic or phonemic criteria within time constraints (11–13). Successful performance on the VFT relies critically on phonemic fluency and executive control under time pressure, and depends on the integrity of prefrontal executive control systems and efficient functional connectivity within relevant neural networks (12, 14, 15). Therefore, examining neural activation patterns during VFT performance in children with ASD may provide valuable insight into the neural mechanisms underlying their characteristic deficits in phonemic fluency and executive control (16, 17).

Despite a growing body of neuroimaging research demonstrating atypical neural activation and disrupted functional connectivity during EF tasks in individuals with ASD (18–21), three key knowledge gaps remain. First, the precise nature of cortical activation alterations in core brain regions during VFT performance remains unclear, with ambiguity about whether these changes reflect hyperactivation, hypoactivation, or atypical activation patterns (22). Second, prior research investigating task-modulated functional connectivity in ASD has produced inconsistent findings, often limited by methodological constraints (23). Third, relatively few investigations have adopted an integrative framework that simultaneously examines both localized neural responses and distributed network connectivity during task execution, despite converging theoretical models emphasizing their interaction as a fundamental mechanism underlying executive dysfunction in ASD (24). Importantly, the clinical relevance of these neural abnormalities remains insufficiently characterized, particularly concerning their relationship with core symptoms of autism. Clarifying this relationship could yield critical mechanistic insights and facilitate the identification of potential biomarkers.

Functional near-infrared spectroscopy (fNIRS) provides distinct methodological advantages for developmental neuroimaging research, particularly its tolerance to head movement and its compatibility with naturalistic experimental paradigms that permit verbal responses (25–28). These advantages make fNIRS especially well-suited for investigating cortical activation during active language production tasks, such as the VFT, in pediatric populations, including children with ASD.

Leveraging these technical strengths, the current study used fNIRS to examine cortical function in children with ASD, using an age-, gender-, and FSIQ-matched control group of children with ID. This design enabled us to address three key research objectives. The first objective was to determine whether children with ASD (FSIQ < 70) exhibit distinct patterns of cortical activation, particularly in prefrontal regions, during the VFT compared to ID controls. The second objective was to investigate potential group differences in the strength of functional connectivity within critical neural networks, with a specific focus on prefrontal-temporal interactions. The third objective was to explore whether atypical neural activation patterns in the ASD cohort correlate with the severity of clinical symptoms. The core methodological rationale for selecting an ID control group rather than typically developing children lies in the careful matching of cognitive ability (FSIQ < 70), which allows us to dissociate generalized neurodevelopmental effects associated with intellectual impairment from neural mechanisms potentially specific to the core ASD symptoms, such as language and executive dysfunction. This approach provides a clearer window into the neural signature of ASD.

By simultaneously analyzing regional brain activation and interregional functional connectivity during VFT performance alongside clinical symptomatology, this study aims to elucidate the neural correlates of language and EF impairments in ASD. Demonstrating significant associations between specific cortical abnormalities (e.g., prefrontal hypoactivation) and elevated scores on the Childhood Autism Rating Scale (CARS) would offer crucial evidence of brain-behavior relationships. Such findings would not only enhance the mechanistic understanding of ASD pathophysiology but also contribute to the identification of promising neural targets for therapeutic intervention.

## Materials and methods

### Subjects

Participants met the following inclusion criteria: children aged 6–12 years who had received a formal diagnosis of ASD according to DSM-5 criteria and who completed the CARS assessment, with clinically stable symptoms (29, 30). Exclusion criteria included: (1) ID (FSIQ ≥ 70); (2) comorbid Attention-Deficit/Hyperactivity Disorder; or (3) symptoms attributable to known genetic or metabolic disorders. All participants underwent a comprehensive neuropsychological evaluation, including cognitive assessment using the Wechsler Intelligence Scale for Children, Fourth Edition (WISC-IV), as well as standardized measures of ASD symptom severity. Based on diagnostic status and cognitive functioning (FSIQ < 70), participants were stratified into two matched groups: an ASD group (n = 33) and an ID group (n = 21). Inclusion criteria for the ID group was as follows: (1) a diagnosis of ‘Unspecified ID’ according to the 10th Revision of the World Health Organization’s International Classification of Diseases (ICD-10); (2) FSIQ < 70 confirmed by WISC-IV; (3) no clinical diagnosis of ASD and CARS scores below the validated cutoff for autism spectrum disorder; and (4) absence of other neurodevelopmental, genetic, or metabolic disorders. Thus, the ID group represents a clinically common phenotype of non-syndromic ID with unspecified etiology. This group served as a reference benchmark matched for overall cognitive level but without the typical social communication deficits of ASD, thereby facilitating the identification of ASD-specific neural activity characteristics. All assessments were conducted by a psychotherapist trained in autism diagnosis and administration of the WISC-IV, ensuring diagnostic reliability. The final cohort comprised 54 well-characterized participants recruited from Shaanxi Provincial Rehabilitation Hospital between October 2024 and June 2025. The study protocol was approved by the institutional review board, and written informed consent was obtained from the parents or legal guardians of all participants before enrollment.

### VFT

VFT was administered during daytime sessions using a standardized protocol. The paradigm consisted of three sequential phases: (1) a 30 s pre-task baseline period, during which participants performed simple numerical counting (repetitively counting numbers 1–5); (2) a 60 s active task phase requiring generative language production; and (3) a 60 s post-task recovery baseline, during which the same counting procedure was repeated. The selection of target Chinese characters was guided by several considerations. First, they were all common single-character words typically acquired during the early school years. Second, modern Chinese word frequency dictionaries were consulted to ensure that all three were of high-frequency characters (approximate word frequencies: 白 [rank ~150], 北 [rank ~200], 大 [rank ~10]), thereby minimizing potential confounding effects of lexical familiarity. Finally, the characters were comparable in visual complexity, with a similar and relatively small number of strokes (白: 5 strokes; 北: 5 strokes; 大: 3 strokes), reducing variability in cognitive load related to visual complexity or writing difficulty. The character sets were systematically rotated every 20 s throughout the task to maintain cognitive engagement and minimize response latency.

### Behavioral data recording and analysis

During the 60 s active task phase, participants were instructed to generate as many valid two-word fixed phrases or compound words as possible, using one of the three sequentially presented Chinese characters (白, 北, 大) as the first character of each phrase. Each target character was presented for a 20 s block. A response was considered valid and on-topic only if it simultaneously met the following criteria: (1) it constituted a commonly used and meaningful two-character fixed expression in Mandarin Chinese; (2) the target Chinese character appeared as the first character of the expression. For example, for the character ‘白’ (white), valid responses included白云 (white cloud) and 白天 (daytime); for ‘北’ (north), 北方 (north direction) and北京 (Beijing); for ‘大’ (large), 大人 [adult] and 大小 (size). Invalid responses included repeated phrases, meaningless combinations (e.g., ‘白书’ [white book]), or expressions in which the target character did not appear in the first position (e.g., ‘雪白’ [snow-white] to the target ‘白’). All verbal responses were audio-recorded and transcribed verbatim. Scoring was performed independently by two trained raters who were blinded to group membership. Inter-rater reliability was excellent (Cohen’s κ = 0.92). Discrepancies, which occurred in fewer than 5% of responses, were resolved through consensus discussion with a third senior researcher. Character sets were systematically rotated every 20 s to maintain cognitive engagement and minimize response latency.

### fNIRS measurement

**Figure 1:**
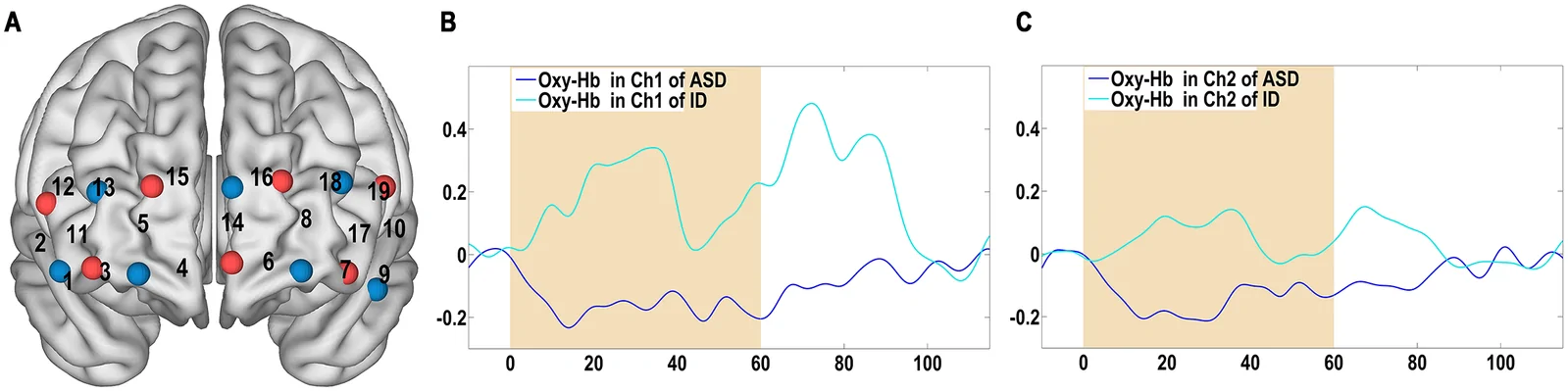
**(A)** The position of 19 NIRS channels. **(B, C)** Time courses of mean Oxy-Hb changes of Ch1 and 2. Averaged individual waveforms of changes in Oxy-Hb concentration in each channel during the VFT in the ASD and ID groups. Ch: channel.

Participants were seated comfortably in a quiet room during the fNIRS recording. Hemoglobin responses were measured using a multi-channel near-infrared optical imaging system (NirSmart-6000A equipment, Danyang Huichuang Medical Equipment Co., Ltd., China). The sampling rate was 11 Hz, and light wavelengths of 730 nm and 850 nm were used, with 808 nm used as an isotopic reference. A total of 14 SD probes, consisting of 7 sources and 7 detectors, were arranged with a fixed inter-probe distance of 3 cm to cover the bilateral prefrontal cortex (PFC) and temporal cortices, yielding 19 fNIRS measurement channels. Channel localization was defined as follows: Channels 1, 2, 9, and 10 corresponded to Inferior frontal gyrus (IFG); Channels 3–8, 11, and 13–18 covered the frontopolar cortex (FPC); Channel 10 itself corresponded to the pars triangularis of Broca’s area; and Channels 12 and 19 represented the dorsolateral prefrontal cortex (DLPFC) (**Figure 1A**). For anatomical registration, the three-dimensional (3D) coordinates of each source and detector were recorded using a Polhemus Patriot digitizer (Polhemus, USA). These coordinates were subsequently co-registered to the Montreal Neurological Institute (MNI) standard brain space using a template provided within the NirSpark software. Based on the resulting MNI coordinates, each channel was assigned to its corresponding Brodmann area and anatomical label (e.g., IFG, DLPFC) with reference to the Automated Anatomical Labeling (AAL) atlas, ensuring spatial comparability across participants and studies. Most inpatients completed the fNIRS assessment within three days of hospital admission. The MNI coordinates and corresponding anatomical labels for each channel are provided in **Supplementary Table 6**.

### Data processing and analysis

fNIRS data preprocessing was conducted using the NirSpark analytical platform (HuiChuang, China). Motion artifacts were corrected through a two-stage procedure: first, detection of signal deviations via moving standard deviation analysis; second, reconstruction of corrupted segments using cubic spline interpolation. Physiological noise was minimized by applying a bandpass filter (0.01–0.20 Hz) to remove cardiopulmonary signals and low-frequency artifacts. Optical density data were subsequently converted to hemoglobin concentration changes using the modified Beer-Lambert law. Oxy-hemoglobin (Oxy-Hb) was chosen as the primary indicator due to its higher signal reliability (31). Channels were excluded if the original signal strength fluctuated beyond the device’s dynamic range or if the signal-to-noise ratio was below 2. These criteria ensured adequate data quality for group comparison. A total of 59 participants were initially recruited. Among these, three were excluded for excessive head motion during the task (defined as more than 50% of channels marked as bad), and two were excluded for incomplete VFT task performance. The final dataset comprised 33 children with ASD and 21 children with ID, who were included in all statistical analyses. Task-related hemodynamic responses were analyzed within a 125 s time window, including a 10 s pre-task baseline, a 60 s active VFT phase, and a 55 s post-task recovery phase. The active phase served as the primary period for quantifying mean Oxy-Hb changes, with baseline correction performed via linear regression. Hemodynamic response waveforms were then averaged across all 19 channels for each group.

To mitigate individual variability in probe and channel placement due to differences in head size, mean activation values were computed for channels grouped by Brodmann areas. Based on 3D coordinate localization (Polhemus, USA) and Brodmann mapping, the 19 channels were grouped into two distinct brain regions (32, 33): 1) Inferior frontal gyrus (IFG) region, corresponding to Brodmann areas 44/45, which included Channels 1, 2, 9, and 10 (with Channel 10 specifically located in the pars triangularis of Broca’s area).; (2) Dorsolateral prefrontal and frontopolar cortex (DLPFC/FPC) region, which encompassed all remaining channels: Channels 3–8, 11, and 13–19. Within this region, Channels 12 and 19 corresponded to the DLPFC (Brodmann areas 9/46), while Channels 3–8, 11, and 13–18 corresponded to the FPC (Brodmann area 10). This grouping was designed to merge spatially adjacent and functionally related channels for regional-level statistical analyses, while all channel-level comparisons reported in this study (e.g., group differences in activation and connectivity, correlations with clinical variables) were performed at the full spatial resolution to preserve localized effects.

Functional connectivity was assessed using the preprocessed Oxy-Hb time series during the entire 60 s task period. To reduce confounding from head motion and physiological noise, the same preprocessing steps used for activation analysis were applied, including motion artifact correction via moving-standard-deviation detection and cubic-spline interpolation, followed by band-pass filter (0.01–0.20 Hz) to attenuate cardiopulmonary oscillations and low-frequency drift.

For each participant, pairwise Pearson correlation coefficients (or Spearman’s correlations where appropriate) were calculated between the time series of all 19 channels, yielding a 19 × 19 correlation matrix. Global signal regression (GSR) was not applied. For each participant, pairwise Pearson correlation coefficients were calculated between all 19 channels, followed by Fisher Z-transformation. Global connectivity was defined as the mean of absolute Z-transformed correlation values, reflecting overall connection strength without distinguishing direction. To verify the effectiveness of our preprocessing in suppressing non-neural signals, we performed a sensitivity analysis comparing each channel’s time series before and after filtering with concurrently recorded heart rate (when available) or standard physiological noise templates. Correlations with physiological signals were reduced to negligible levels after filtering, supporting the neurophysiological specificity of the final connectivity measures.

All correlation coefficients were Fisher Z-transformed to improve normality for group-level analyses. Mean connectivity strength for each participant was defined as the average of the absolute Z-transformed values across all unique channel pairs. Group differences in functional connectivity were initially examined using independent two-sample t-tests for each of the 171 Fisher Z-transformed correlation coefficients (channel pairs). To control for multiple comparisons while balancing sensitivity and false-positive risk in this exploratory network-level analysis, *p*-values were corrected using the false-discovery-rate (FDR) procedure across all 171 tests.

Normality of continuous variables was assessed using the Shapiro-Wilk test. Between-group comparisons of continuous variables were performed using independent two-sample t-tests or Mann–Whitney U tests, as appropriate, while categorical variables were analyzed using chi-square tests. Associations between Oxy-Hb concentrations and clinical variables were examined using Spearman correlation analysis. The discriminatory performance of Oxy-Hb measures was evaluated using receiver operating characteristic (ROC) analysis. All statistical analyses were conducted in SPSS 27.0 (IBM Inc., Chicago, IL, USA), and figures were generated using Adobe Photoshop and OriginPro (OriginLab Corporation, USA). To control for Type I error in mass-univariate analyses, the Benjamini--Hochberg FDR procedure was applied separately to two sets of tests: (1) the 19 channel-wise comparisons of mean Oxy-Hb changes between groups (**Table 1**), and (2) the 171 pairwise channel-to-channel connectivity comparisons (corresponding to the full connectivity matrix). An FDR-corrected *p* value < 0.05 was considered statistically significant for each set of tests.

**Table 1:** Demographic and clinical characteristics (mean ± SD).

	ASD (n=33)	ID (n=21)	t/Chi-square value	p
Demographics				
Age (years)	8.21 ± 2.27	9.05 ± 1.40	-1.509	0.137
Gender (male/female)	30/3	16/5	2.203	0.138
delivery mode (yes/no)	21/12	13/8	0.017	0.898
Parental care (yes/no)	24/9	11/10	2.330	0.127
Being an only child (yes/no)	19/14	13/8	0.100	0.752
Clinical characteristics				
Wechsler Children’s Intelligence Test-IV				
Full-Scale Intelligence Quotient (FSIQ)	58.55 ± 6.41	58.00 ± 4.32	0.343	0.733
Childhood Autism Rating Scale (CARS)	34.85 ± 2.32	NA	NA	NA
VFT Behavioral Performance				
Correct Words/Minute	1.45 ± 0.506	1.76 ± 0.83	-1.693	0.096
Perseverations (count)/Minute	0.42 ± 0.502	0.38 ± 0.498	0.310	0.758

Delivery mode refers to whether the participant was born via cesarean section or vaginal delivery. This variable was included to provide demographic characterization and to ensure group comparability on basic perinatal factors.

## Results

### Participant characteristics

**Table 2** summarizes the demographic and cognitive profiles of participants according to diagnosis and FSIQ. The ASD group (FSIQ < 70) consisted of 33 children (30 males, 3 females) with a mean age of 8.21 years (SD = 2.27). The ID group (FSIQ < 70) comprised 21 children (16 males, 5 females) with a mean age of 9.05 years (SD = 1.40). Intergroup comparisons revealed no significant differences in age (t = -1.509, *p* = 0.137), gender distribution (χ² = 2.203, *p* = 0.138), delivery mode (χ² = 0.017, *p* = 0.898), parental care status (χ² = 2.330, *p* = 0.127), or only-child status (χ² = 0.100, *p* = 0.752). The groups were also matched on FSIQ, with a mean score of 58.55 (SD = 6.41) for the ASD group and 58.00 (SD = 4.32) for the ID group (*p* = 0.733). On the CARS, the ASD group had a mean score of 34.85 (SD = 2.32).

**Table 2:** Differences in oxygenated hemoglobin concentration during two sets of channel-based task states. .

Channel	S-D	ASD group	ID group	t	p (FDR-corrected)
1	S2-D2	-0.1588	0.1753	-4.272	**0.002**
2	S2-D7	-0.1418	0.0480	-3.827	**0.003**
3	S3-D2	0.0133	0.0350	-0.279	0.963
4	S3-D3	0.0263	-0.0119	0.608	0.873
5	S3-D8	-0.0889	-0.0205	-1.157	0.800
6	S4-D3	0.0310	0.0347	-0.046	0.963
7	S4-D4	-0.0139	0.0090	-0.312	0.963
8	S4-D9	-0.0455	-0.0095	-0.644	0.873
9	S5-D4	0.0519	0.0714	-0.148	0.963
10	S5-D10	0.0190	0.0311	-0.126	0.963
11	S8-D2	-0.0511	0.0769	-2.221	0.195
12	S8-D7	-0.0539	0.0336	-1.161	0.800
13	S8-D8	-0.1025	-0.0099	-1.836	0.342
14	S9-D3	-0.0579	-0.0149	-0.750	0.873
15	S9-D8	-0.1369	-0.0706	-0.735	0.873
16	S9-D9	-0.0134	0.0417	-0.604	0.873
17	S10-D4	0.0080	0.0448	-0.600	0.873
18	S10-D9	0.0138	0.0308	-0.276	0.963
19	S10-D10	0.0106	0.0026	0.098	0.963

P values less than 0.05 are shown in bold.

### Behavioral performance on the VFT

Behavioral analyses confirmed that both groups were engaged in the task. There were no significant differences between the ASD and the ID groups in correct word generation per minute (ASD: mean ± SD = 1.45 ± 0.506; ID: 1.76 ± 0.83; t = -1.693, *p* = 0.096) or perseverations per minute (ASD: mean ± SD = 0.33 ± 0.479; ID: 0.38 ± 0.498; t = -0.351, *p* = 0.727). These results indicate similar verbal output levels between groups; however, marked floor effects were observed in both groups, limiting the interpretability of behavioral differences. The overall low rate of phrase generation (~1.5 valid phrases per minute) indicates substantial floor effects in both groups, consistent with the high cognitive demands of the task in children with FSIQ < 70.

### Mean Oxy-Hb changes during the VFT

Cortical hemodynamic responses were measured using fNIRS across 19 predefined VFT during the VFT, with channel locations depicted in **Figure 1A**. Statistical analyses revealed significant between-group differences in mean Oxy-Hb changes in prefrontal channels. No subjective interpretations of waveform shape, slope, or peak morphology were used; all inferences were based on quantitative group comparisons.

Hemodynamic responses were quantified using mean Oxy-Hb changes during the task period, with group comparisons performed using statistical tests. Quantitative analyses confirmed significantly reduced Oxy-Hb activation in the ASD group within two prefrontal channels (Channels 1 and 2), anatomically corresponding to the IFG (**Figures 1B, C**). These differences in cortical activation persisted throughout the entire task duration. Full hemodynamic waveforms of Channels 3–19 during the task are presented in **Supplementary Figure 1**.

**Figure 2:**
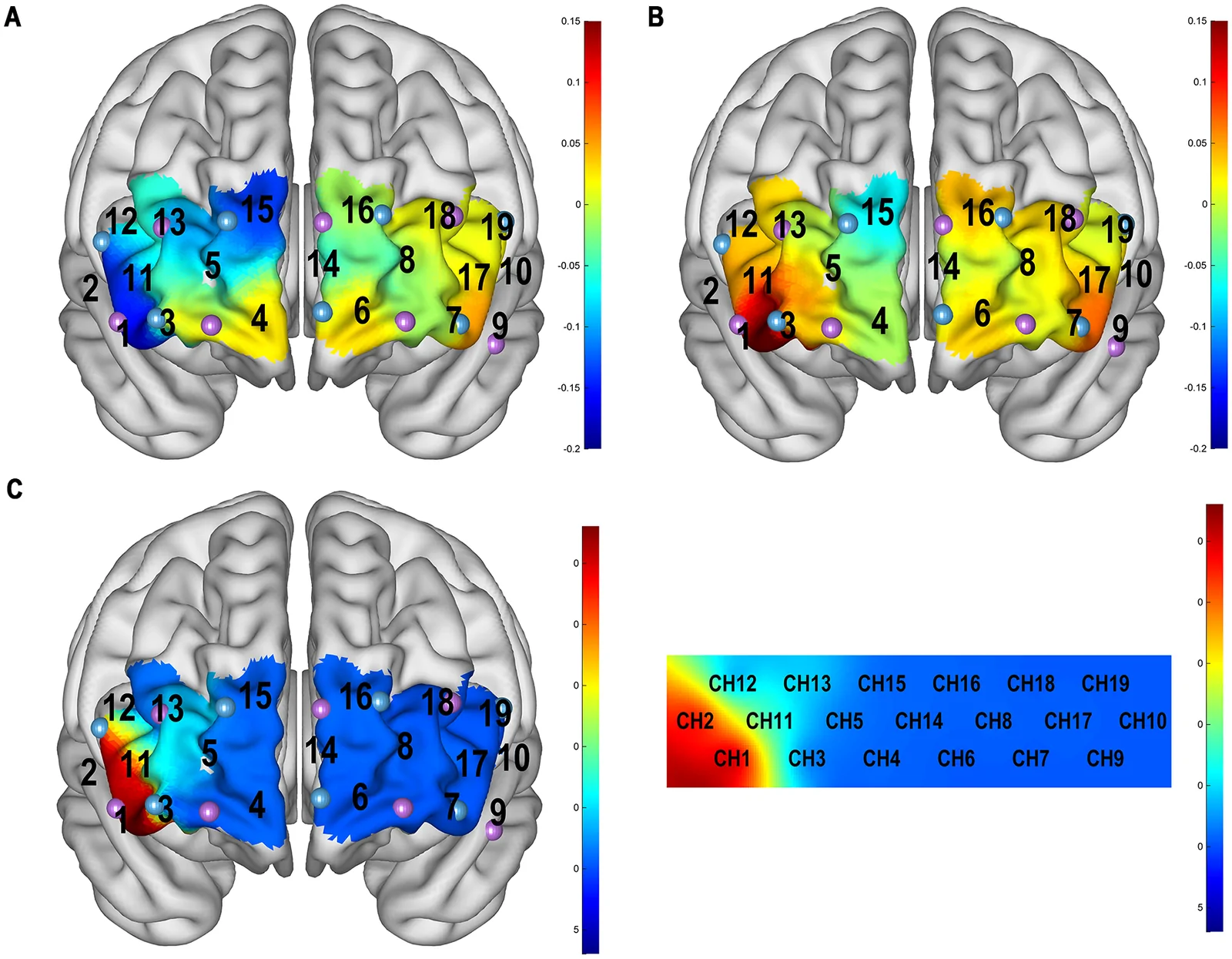
**(A)** The mean Oxy-Hb changes in each channel of the ASD group. **(B)** The meaning Oxy-Hb changes in each channel of the ID group. **(C)** *p*-values of each channel activation for the ASD group compared to the ID group.

Statistical testing of mean Oxy-Hb changes revealed significant between-group differences in PFC activation (**Figure 2**, **Table 1**). After FDR correction, inter-group differences remained statistically significant only in Channel 1 (t = -4.272, FDR-corrected *p* = 0.002) and Channel 2 (t = -3.827, FDR-corrected *p* = 0.003) both corresponding to IFG.

### Functional connectivity

**Figure 3:**
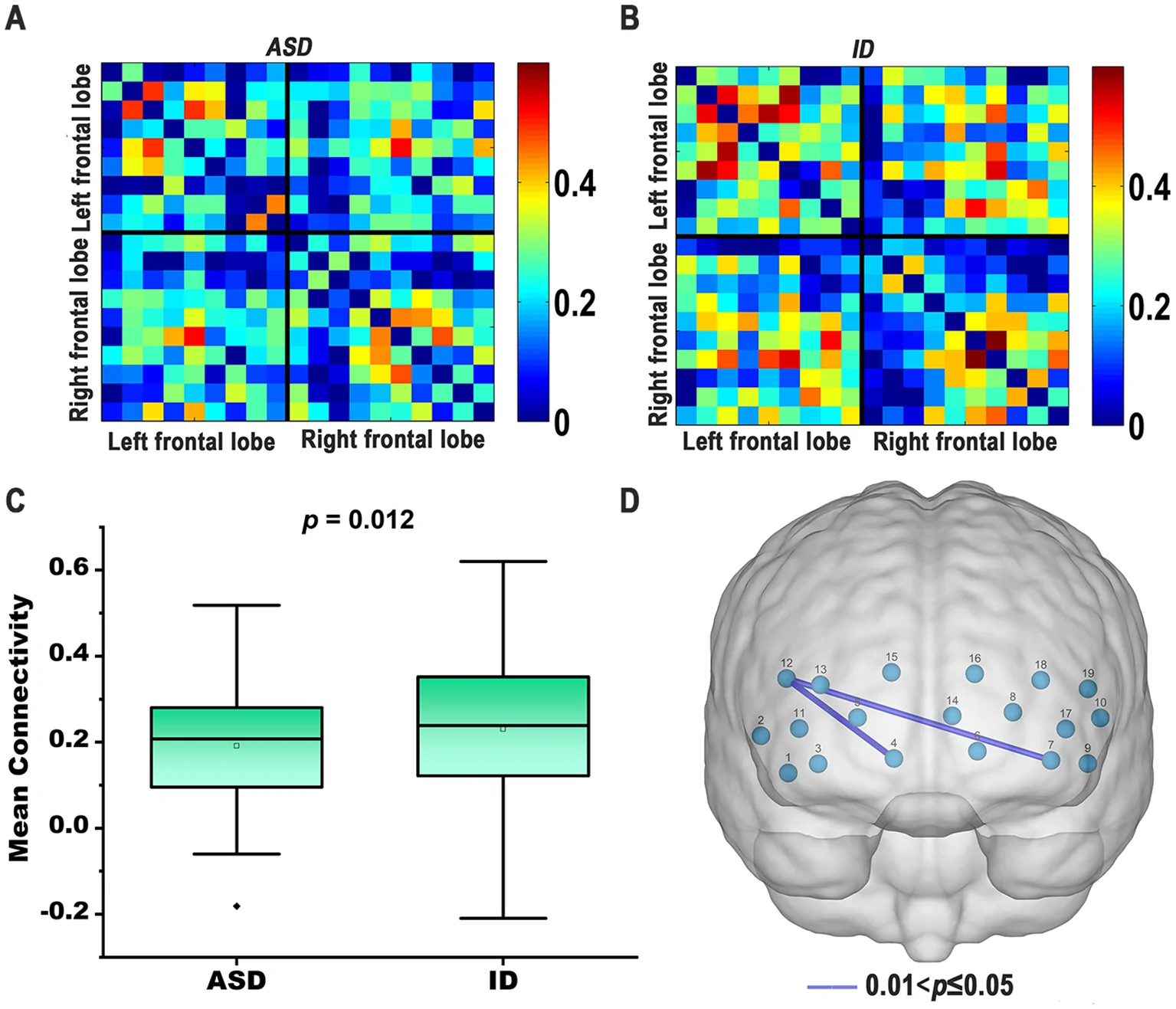
**(A, B)** Connectivity between the hemodynamic response of 19 channels. Left frontal lobe: Channels 6–10 and 16-19; Right frontal lobe: Channels 1–5 and 11-15. **(C)** Violin plots showing the distribution of global mean connectivity strength (average of absolute Z-values across all channel pairs) for each group. The ASD group showed significantly lower mean connectivity (*p* < 0.001). **(D)** Schematic representation of the fNIRS channel configuration on the prefrontal cortex. Lines connecting channels indicate the two channel pairs (4–12 and 7–12) that showed significantly enhanced connectivity in the ASD group after FDR correction for all 171 pairwise tests (corrected *p* < 0.05). To ensure visualization clarity, only statistically significant differences are highlighted.

Functional connectivity analysis produced 19×19 correlation matrices for each experimental group (ASD: **Figure 3A**; **ID**: **Figure 3B**; **Supplementary Tables 1**, **S2**). At the global network level, children with ASD demonstrated a trend toward reduced mean connectivity strength relative to ID controls, with average values of 0.191 (M = 0.191,SD = 0.130) and 0.230 (M = 0.230, SD = 0.155), respectively (*p* < 0.001; **Figure 3C**).

After FDR correction across all 171 unique channel-pairwise comparisons, significant group differences were observed for two channel pairs only, specifically those connecting Channels 4 and 12 and Channels 7 and 12 (corrected *p* < 0.05; **Table 3** and **Figure 3D**). Importantly, both significant connections involved Channel 12, which is anatomically located in the right DLPFC. Full statistical details, including uncorrected and FDR-corrected *p*-values for all channel pairs, are provided in **Supplementary Table 3**.

**Table 3:** Channel pairs with significant differences in connectivity strength between groups (mean ± SD).

Channel to channel	Connectivity strength (M ± SD)		t	p (FDR corrected)
	ASD group	ID group		
4~12	0.34 ± 0.46	-0.20 ± 0.51	4.0781	**0.0134**
7~12	0.32 ± 0.46	-0.21 ± 0.44	4.1945	**0.0134**

P values less than 0.05 are shown in bold.

Notably, despite the observed pattern of global differences in overall connectivity strength, connectivity strength within these two specific channel pairs (4–12 and 7–12) was significantly greater in the ASD group than in the ID group (**Table 3**). This suggests a localized alteration in functional coupling involving the DLPFC in children with ASD. For comparison, the ID group exhibited moderate and spatially distributed functional connectivity across prefrontal channels, with no localized hyperconnectivity involving the DLPFC.

### Correlations and regression analyses with clinical characteristics

**Figure 4:**
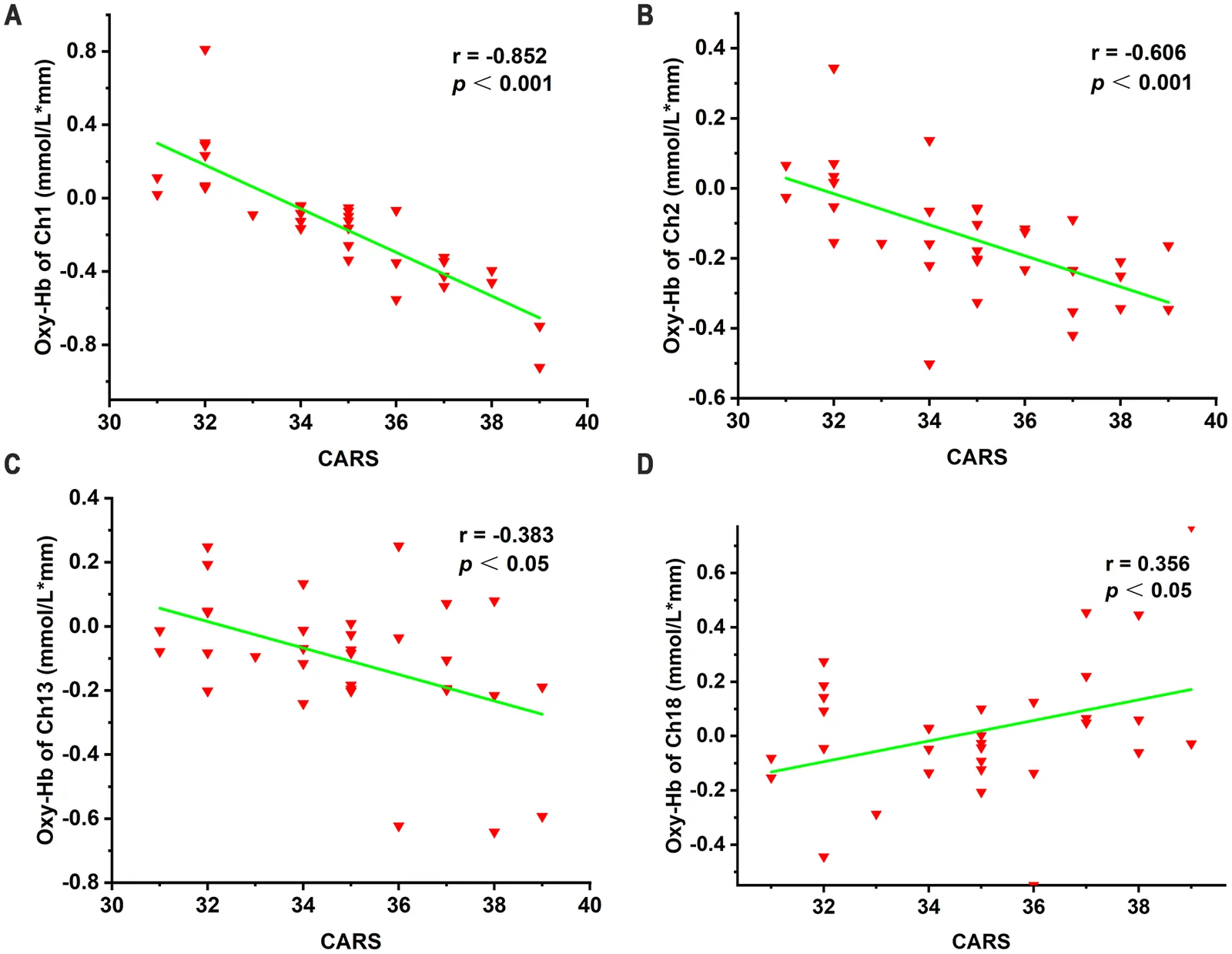
Correlation between CARS scores and mean Oxy-Hb changes in Channels 1, 2,13 and 18.

Bivariate correlation analyses using Spearman’s rank correlation were first conducted to examine the relationship between CARS total scores and mean Oxy-Hb changes in specific prefrontal channels (**Table 4**, **Figure 4**). Specifically, strong negative correlations were observed in Channel 1 (r = -0.852, *p* < 0.001) and Channel 2 (r = -0.606, *p* < 0.001), both corresponding anatomically to the IFG, as well as in Channel 13 (r = -0.453, *p* < 0.01). In contrast, Channel 18 exhibited a significant positive correlation with CARS scores (r = 0.356, *p* < 0.05). This finding should be considered preliminary and requires replication in future studies.

**Table 4:** Associations between channel Oxy-Hb changes and clinical characteristics in the ASD group.

Channel	Childhood autism rating scale (CARS)	
	r/rs	p
1	-0.852	**<0.001**
2	-0.606	**<0.001**
13	-0.453	**0.008**
18	0.356	**0.042**

P values less than 0.05 are shown in bold.

To determine whether these relationships were independent of potential confounding factors, we conducted a series of multiple linear regression analyses (**Table 5**). Regression models were initially explored across all 19 channels to identify prefrontal regions in which task-related Oxy-Hb changes were significantly associated with autism symptom severity, as indexed by CARS scores, after adjustment for covariates (demographics, task performance, and FSIQ). This screening procedure indicated that only Channels 1, 2, and 13 exhibited significant associations with CARS scores (p < 0.05) and were therefore retained for final model reporting. Although Channel 18 showed a bivariate correlation with CARS, this relationship did not demonstrate a significant independent relationship in the regression framework and was therefore excluded from multivariable analyses. Based on these results, three separate models were constructed, with mean Oxy-Hb changes during the VFT in Channel 1 (Model 1), Channel 2 (Model 2), and Channel 13 (Model 3) specified as the dependent variables, respectively. In each model, the CARS total score was entered as the primary predictor, while demographic variables, VFT behavioral performance, and FSIQ were included as covariates.

**Table 5:** Multiple linear regression models predicting neuroimaging data from demographic and clinical characteristics.

Indicator	Variable	Model 1		Model 2		Model 3		VIF
		ß	t	ß	t	ß	t	
Independent variable	Constant		4.537		8.075		1.946	
	Age (years)	0.047	0.445	0.089	0.533	-0.217	-1.417	1.253
	Gender (male/female)	-0.111	-0.975	-0.198	-1.099	-0.307	-1.870	1.446
	delivery mode (yes/no)	-0.097	0.067	-0.158	-0.990	0.000	-0.002	1.141
	Parental care (yes/no)	-0.035	-0.329	0.016	0.096	0.273	1.764	1.284
	Being an only child (yes/no)	0.086	0.709	0.017	0.091	-0.005	-0.032	1.634
	Correct Words/Minute	-0.062	-0.611	0.053	0.329	0.039	0.268	1.158
	Perseverations (count)/Minute	0.222	2.044	0.165	0.958	0.313	1.997	1.321
	FSIQ	-0.020	-0.179	-0.224	-1.249	-0.099	-0.609	1.042
	CARS	-0.901***	-9.148	-0.670**	-4.289	-0.454**	-3.188	1.134
	R value	0.891		0.696		0.756		
	R² value	0.795		0.484		0.571		
	R²change value	0.714		0.282		0.404		
	F value	9.894***		2.397***		3.408**		

Note: Refer to level 0.05, where ***p < 0.001, **p ≤ 0.05

Across all three models, autism symptom severity emerged as a robust and independent predictor of prefrontal hemodynamic responses. As shown in **Table 5**, higher CARS scores were consistently associated with reduced Oxy-Hb changes in Model 1 (β = -0.901, *p* < 0.001), Model 2 (β = -0.670, *p* < 0.05), and Model 3 (β = -0.454, *p* < 0.05), even after accounting for the covariates. In contrast, demographic and behavioral covariates did not exhibit significant independent effects in the neural measures in these models (all *p* > 0.05; **Table 5**), supporting the specificity of the association between prefrontal hemodynamics and core ASD symptoms.

## Discussion

In the present study, fNIRS was used to investigate cortical activation patterns during a VFT in children with ASD and ID controls. The results revealed significant group differences in PFC activation and in large-scale network integration, which corresponded to variations in phonemic fluency and executive control under time pressure among participants. Although the VFT is a production task, its execution critically depends on semantic retrieval and executive control. Thus, activation patterns during the VFT primarily reflect the efficiency of language production–related semantic processing and executive control systems, which are core components of expressive language function.

Comparative analysis focusing on children with FSIQ below 70 demonstrated a significant reduction in prefrontal activation in the ASD group when compared with ID controls. In particular, attenuated hemodynamic responses were observed in Channels 1 and 2, anatomically localized to right IFG. The left IFG, in particular, is widely recognized as a highly integrated cortical hub that supports high-order cognitive integration, with a well-established role in articulated speech production. Conversely, the right IFG is more critically involved in behavioral regulation, particularly through the inhibition of prepotent responses. This dual involvement indicates that IFG-mediated cognitive control processes serve a critical two-fold purpose, enabling the selection and fluent production of context-appropriate verbal output while simultaneously suppressing competing or irrelevant information and actions (34). The present findings are consistent with prior evidence demonstrating the critical role of the IFG in language processing and EF (35–37), suggesting that altered activation in this region may contribute to core ASD symptoms. Moreover, the observed pattern of prefrontal hypoactivation aligns with previous reports in children with ASD during cognitive tasks (38–40).

The verbal fluency task requiring fixed phrases, as implemented in this study, engages core processes of phonemic fluency and executive control under time pressure, which rely on the integrity of prefrontal regulatory systems. given that the retrieval of overlearned phrase chunks demands strategic monitoring and search processes—functions supported by the dorsolateral prefrontal cortex (DLPFC) and frontopolar cortex (BA 10) (45, 46). The predominantly right-lateralized prefrontal activity observed in our ASD group may reflect a compensatory mechanism within executive control networks to sustain task performance (47). Importantly, this observation does not undercut the task’s relevance to language difficulties in ASD; instead, it suggests that language production impairments could stem from inefficient engagement of broader executive and memory networks essential for fluent communication, rather than being confined to dysfunction in core language regions (48). The pattern of significantly reduced task-evoked oxy-hemoglobin concentration changes in the right inferior frontal gyrus (Channels 1 and 2) coupled with enhanced right DLPFC–FPC connectivity (Channel pairs 12–4 and 12–7)in the ASD group aligns with this interpretation, indicating that children with ASD rely on alternative neural pathways to attain comparable behavioral output. These neural characteristics were significantly associated with autism symptom severity (CARS scores), underscoring their relevance to the core pathophysiology of ASD.

Functional connectivity analyses further demonstrated significantly reduced neural network integration in children with ASD compared with FSIQ-matched ID controls. Quantitative indices indicated weaker overall interregional connectivity in the ASD group during the VFT, consistent with previous reports of atypical connectivity patterns in ASD (40–43). This global pattern of hypoconnectivity suggests reduced efficiency in information exchange across distributed networks supporting language and EF.

Notably, a more complex picture emerged when examining specific channel pairs. In contrast to the overall trend, functional coupling between Channel 12, corresponding to the DLPFC, and Channels 4 and 7, which primarily cover the FPC, was significantly stronger in the ASD group (**Table 3**). This localized increase in connectivity, occurring alongside global hypoconnectivity, points to specific alterations within the prefrontal executive network.

This seemingly paradoxical pattern may be explained by several non-mutually exclusive mechanisms. Enhanced coupling between the DLPFC, a key region involved in lexical retrieval and cognitive control (44), and the FPC may reflect compensatory neural recruitment. To sustain task performance, the DLPFC may engage in stronger and less selective interactions with broader frontal regions to offset deficient integration within core language and executive circuits. Alternatively, this localized hyperconnectivity may reflect increased cognitive load or the adoption of atypical strategies, such as heightened reliance on working memory and sustained monitoring, in children with ASD, directly manifesting as heightened local synchronization. Such an interpretation is consistent with models depicting ASD as a disorder of complex network imbalance, featuring local over-integration concurrent with broader under-connectivity (45).

Therefore, the connectivity profile observed in children with ASD is characterized by a dual anomaly: a widespread reduction in network-level integration combined with a specific increase in connectivity between the DLPFC and frontal polar regions. This “dorsal-polar” hyperconnectivity likely does not represent more efficient function but may instead be a compensatory or epiphenomenal feature of a dysregulated network. These findings indicate that children with ASD not only exhibit insufficient regional activation (e.g., in the IFG) but also show impairments in the efficient and flexible dynamic configuration of prefrontal executive networks. This impaired network orchestration may underlie their reduced neural efficiency despite comparable behavioral output.

Importantly, no significant differences were found between the ASD and ID groups in explicit behavioral output on the VFT, including correct words per minute and perseverations per minute. Although statistically non-significant, there was a trend indicating that children with ID produced more correct words per minute than those with ASD (1.76 vs. 1.45 words/minute), with a small–to-moderate effect size (Cohen’s d = 0.46). This trend suggests a slight advantage for children with ID in verbal production during this task. However, the key point is that, despite this potential behavioral production trend, the ASD group exhibited more marked reductions in prefrontal activation and functional connectivity. Neural group differences were observed despite similar behavioral output, but these observations should not be interpreted as reflecting equivalent task engagement or processing effort. Instead, it points to fundamental differences in neural resource allocation during phonemic fluency and executive control performance. The fact that both groups exhibited similarly low behavioral output highlights that the task imposes high cognitive demands on both ASD and ID children. The presence of significant neural differences despite comparable (or even slightly lower trend in ASD) behavioral performance in the ASD group further supports the notion that impaired neural processing efficiency is an intrinsic feature rather than simply due to differences in task engagement or ability.

The comparable behavioral performance observed between groups, despite reduced prefrontal activation and altered connectivity in children with ASD, suggests differences in neural processing efficiency or the engagement of compensatory mechanisms. Children with ASD may achieve similar verbal output by exerting increased cognitive effort or relying on alternative neural pathways, exemplified by the observed hyperconnectivity between the DLPFC and frontal polar regions. This pattern aligns with the ‘neural efficiency’ hypothesis, which posits that individuals with ASD may require greater regional coordination or sustained attentional control to perform tasks that typically developing neural networks accomplish with less integrated activation.

A central finding of this study is that the severity of core autism symptoms, as measured by CARS scores, serves as an independent and specific predictor of hemodynamic responses in specific prefrontal regions—especially the IFG and Channel 13—even after controlling for general intellectual ability, task performance, and demographic factors.

First, this result strengthens the direct link between prefrontal dysfunction, notably within the IFG, and the core pathophysiology of ASD. While prior research has frequently reported abnormal prefrontal activation in individuals with ASD, disentangling these effects from confounds related to task performance or co-occurring ID has proven challenging. By incorporating an FSIQ-matched ID control group and rigorously adjusting for covariates using multiple regression models, this study provides clear evidence that hypoactivation in the IFG during a VFT is significantly associated with the social-communicative deficits characteristic of ASD. Crucially, this association is independent of overall cognitive functioning. These findings support the hypothesis that executive and language impairments in ASD arise from distinct neural mechanisms, positioning IFG dysfunction as a key node linking the behavioral phenotype of ASD with its underlying neurophysiology.

Second, the observed regional heterogeneity in correlation patterns, featuring both negative versus positive associations, suggests functional differentiation and complex compensatory mechanisms within the PFC in response to symptom severity. Specifically, a consistent negative correlation was found in the IFG (Channels 1 and 2) and adjacent regions (Channel 13), indicating that greater symptom severity corresponds to lower task-related activation. This likely reflects reduced efficiency within this core language and executive network. Conversely, activation in the frontal polar region (Channel 18) exhibited a positive correlation with symptom severity. Although this finding is preliminary and not independently confirmed by the regression analyses, it may reflect an atypical neural resource mobilization strategy. As functional impairment in primary networks such as the IFG increases, individuals may compensate by engaging additional regions like the frontal pole, which is involved in metacognition, monitoring, and compensatory strategies, to sustain task performance. This “dissociation” in activation, characterized by reduced function in core regions alongside increased activity in alternative regions, may represent an important aspect of the heterogeneous neural presentation in ASD.

Several non-mutually exclusive mechanisms may account for this positive association. One speculative explanation is compensatory neural recruitment. Inefficiency within core language and executive networks, such as the IFG, may necessitate the engagement of alternative prefrontal regions to sustain task performance, with greater compensatory effort potentially corresponding to more severe dysfunction in these core networks. However, alternative interpretations warrant equal consideration. The observed activation in this region could also reflect increased task-related cognitive load, heightened performance-related anxiety, excessive self-monitoring, or the processing of ambiguous task demands. These processes may be more pronounced in individuals with greater symptom severity and do not necessarily indicate functional compensation, but rather the additional cognitive cost or atypical engagement strategies required to achieve comparable behavioral output.

Critically, this finding remains exploratory. While it highlights the complexity of prefrontal dysfunction in ASD, it does not allow for definitively distinguish between compensatory, maladaptive, or epiphenomenal neural activity. Future studies using larger cohorts, neuroimaging modalities with higher spatial resolution (e.g., fMRI), and more fine-grained clinical phenotyping are needed to replicate these results and clarify the functional significance of divergent activation patterns within the PFC. Longitudinal designs will be particularly valuable in determining whether these patterns reflect stable traits or dynamic states amenable to intervention.

These present findings also demonstrate the utility of fNIRS as a valuable tool for assessing EF-related brain activity in children with ASD. These results provide meaningful neurobiological insight into the mechanisms underlying executive dysfunction in ASD and highlight potential clinical applications for individualized intervention through modulation of prefrontal activity. Specifically, the identified fNIRS-derived metrics may serve as candidate neural targets for future neurofeedback training, supporting individualized interventions aimed at improving language and EFs in children with ASD.

Several limitations of this study should be acknowledged. First, the relatively small sample size (ASD = 33, ID = 21) may limit the generalizability of the findings and reduce statistical power to detect more subtle group differences, despite the application of FDR correction for multiple comparisons. Second, the cross-sectional design precludes conclusions regarding developmental changes of neural activation patterns. Furthermore, the etiology of ID in the control group was heterogeneous and classified as non-syndromic or unspecified. Although this group was a valuable phenotype matched on overall cognitive level (FSIQ), the inherent etiological heterogeneity implies that participants may have diverse underlying neurofunctional profiles. This variability may introduce confounding variance, making it more challenging to identify neural profiles preferentially associated with ASD from those associated with non-syndromic ID. Future studies using control groups with more specifically defined etiologies, while still controlling for cognitive level, would help to further refine the identification of ASD-associated distinct neurofunctional profile. Methodologically, although fNIRS provides excellent temporal resolution (sampling rate: 11 Hz), its spatial resolution (approximately 30 mm) is lower than that of fMRI, which limits anatomical precision. Future multimodal approaches integrating fNIRS with structural MRI would improve anatomical precision in localizing channel-specific activity. Despite these limitations, the robustness of the present findings is supported by consistent effect sizes observed across multiple prefrontal channels.

Three future research directions emerge from these findings. First, longitudinal studies are needed to characterize developmental trajectories of prefrontal activation patterns in children with ASD. Second, clinical trials could investigate whether neuromodulation interventions targeting the IFG enhance EF and Social interaction ability. Third, multimodal neuroimaging approaches that integrate fNIRS with fMRI (spatial resolution: 1–2 mm) would improve anatomical precision while maintaining the superior temporal resolution of fNIRS (sampling rate: 0.1 s), enabling more EF and Social Communication network-level analyses of language processing in ASD.

These findings highlight an ASD-associated neural profile relevant to executive function differences. Further research is needed to determine clinical implications and potential targets for intervention. In particular, neuromodulatory approaches such as neurofeedback or transcranial stimulation focusing on the IFG and DLPFC warrant further investigation as potential strategies to enhance phonemic fluency and executive control under time in children with ASD.

## Conclusion

In summary, this fNIRS study demonstrates that, during a fixed phrase VFT, children with ASD exhibit a distinct prefrontal neurofunctional profile characterized by reduced activation in the IFG, decreased global functional connectivity, and selectively increased connectivity between the DLPFC and frontal polar regions. Importantly, these neural features were significantly associated with the severity of core autism symptoms, as indexed by CARS scores, and were independent of general intellectual ability. Taken together, the present findings suggest that altered prefrontal network dynamics—may inform future investigations in phonemic fluency and executive control in ASD.

## Data Availability

The raw data supporting the conclusions of this article will be made available by the authors, without undue reservation.

## Supplementary material

Supplementary Figure 1 Full time-series curves of Oxy-Hb concentration changes for Channels 3–19 during the verbal fluency task in ASD and ID groups.
