# Supplementary material for: Altered cortical activation and functional connectivity during fixed phrase verbal fluency in children with autism spectrum disorder
Source: Front Psychiatry. 2026 Aug 14;17:1786619. doi: 10.3389/fpsyt.2026.1786619 (PMC13522225; doi:10.3389/fpsyt.2026.1786619)
Supplement: Supplementary file 1 [file Table1.docx]

**Supplementary Tables**

**Table S1 Data corresponding to brain network connections of ASD.**

|  | **Ch1** | **Ch2** | **Ch3** | **Ch4** | **Ch5** | **Ch6** | **Ch7** | **Ch8** | **Ch9** | **Ch10** | **Ch11** | **Ch12** | **Ch13** | **Ch14** | **Ch15** | **Ch16** | **Ch17** | **Ch18** | **Ch19** |
| --- | --- | --- | --- | --- | --- | --- | --- | --- | --- | --- | --- | --- | --- | --- | --- | --- | --- | --- | --- |
| **Ch1** |  | 0.309 | 0.009 | 0.025 | 0.169 | 0.100 | 0.269 | -0.024 | 0.064 | 0.081 | 0.117 | 0.095 | 0.096 | -0.039 | 0.052 | 0.095 | 0.102 | 0.140 | 0.189 |
| **Ch2** | 0.309 |  | 0.059 | 0.223 | 0.060 | 0.104 | 0.151 | 0.026 | -0.060 | 0.062 | -0.016 | 0.238 | 0.190 | 0.032 | -0.027 | 0.019 | -0.001 | -0.051 | 0.209 |
| **Ch3** | 0.009 | 0.059 |  | 0.345 | 0.279 | 0.266 | 0.332 | 0.214 | 0.266 | 0.221 | 0.373 | 0.207 | 0.433 | 0.108 | 0.150 | -0.022 | 0.257 | 0.145 | 0.314 |
| **Ch4** | 0.025 | 0.223 | 0.345 |  | 0.239 | 0.145 | 0.420 | 0.266 | 0.223 | 0.081 | 0.160 | 0.344 | 0.255 | 0.250 | 0.081 | 0.089 | 0.390 | 0.098 | 0.134 |
| **Ch5** | 0.169 | 0.060 | 0.279 | 0.239 |  | 0.290 | 0.518 | 0.241 | 0.129 | 0.014 | 0.228 | 0.206 | 0.445 | 0.320 | 0.476 | 0.216 | 0.301 | 0.414 | 0.273 |
| **Ch6** | 0.100 | 0.104 | 0.266 | 0.145 | 0.290 |  | 0.238 | 0.448 | 0.170 | 0.169 | 0.186 | 0.051 | 0.282 | 0.122 | 0.102 | -0.182 | 0.210 | 0.074 | 0.229 |
| **Ch7** | 0.269 | 0.151 | 0.332 | 0.420 | 0.518 | 0.238 |  | 0.276 | 0.221 | 0.076 | 0.231 | 0.321 | 0.348 | 0.190 | 0.281 | 0.036 | 0.497 | 0.263 | 0.379 |
| **Ch8** | -0.024 | 0.026 | 0.214 | 0.266 | 0.241 | 0.448 | 0.276 |  | 0.280 | 0.098 | 0.299 | 0.243 | 0.184 | 0.264 | 0.109 | 0.019 | 0.318 | 0.160 | 0.224 |
| **Ch9** | 0.064 | -0.060 | 0.266 | 0.223 | 0.129 | 0.170 | 0.221 | 0.280 |  | 0.140 | 0.261 | 0.253 | 0.240 | 0.116 | 0.035 | 0.150 | 0.410 | 0.271 | 0.360 |
| **Ch10** | 0.081 | 0.062 | 0.221 | 0.081 | 0.014 | 0.169 | 0.076 | 0.098 | 0.140 |  | 0.246 | 0.017 | 0.093 | 0.002 | 0.132 | -0.012 | 0.028 | -0.051 | 0.290 |
| **Ch11** | 0.117 | -0.016 | 0.373 | 0.160 | 0.228 | 0.186 | 0.231 | 0.299 | 0.261 | 0.246 |  | 0.297 | 0.343 | 0.142 | 0.294 | 0.118 | 0.283 | 0.212 | 0.296 |
| **Ch12** | 0.095 | 0.238 | 0.207 | 0.344 | 0.206 | 0.051 | 0.321 | 0.243 | 0.253 | 0.017 | 0.297 |  | 0.328 | 0.306 | 0.096 | 0.115 | 0.241 | 0.267 | 0.208 |
| **Ch13** | 0.096 | 0.190 | 0.433 | 0.255 | 0.445 | 0.282 | 0.348 | 0.184 | 0.240 | 0.093 | 0.343 | 0.328 |  | 0.181 | 0.381 | 0.229 | 0.237 | 0.204 | 0.288 |
| **Ch14** | -0.039 | 0.032 | 0.108 | 0.250 | 0.320 | 0.122 | 0.190 | 0.264 | 0.116 | 0.002 | 0.142 | 0.306 | 0.181 |  | 0.271 | 0.343 | 0.077 | 0.309 | 0.094 |
| **Ch15** | 0.052 | -0.027 | 0.150 | 0.081 | 0.476 | 0.102 | 0.281 | 0.109 | 0.035 | 0.132 | 0.294 | 0.096 | 0.381 | 0.271 |  | 0.290 | 0.074 | 0.247 | 0.143 |
| **Ch16** | 0.095 | 0.019 | -0.022 | 0.089 | 0.216 | -0.182 | 0.036 | 0.019 | 0.150 | -0.012 | 0.118 | 0.115 | 0.229 | 0.343 | 0.290 |  | -0.053 | 0.332 | 0.017 |
| **Ch17** | 0.102 | -0.001 | 0.257 | 0.390 | 0.301 | 0.210 | 0.497 | 0.318 | 0.410 | 0.028 | 0.283 | 0.241 | 0.237 | 0.077 | 0.074 | -0.053 |  | 0.210 | 0.491 |
| **Ch18** | 0.140 | -0.051 | 0.145 | 0.098 | 0.414 | 0.074 | 0.263 | 0.160 | 0.271 | -0.051 | 0.212 | 0.267 | 0.204 | 0.309 | 0.247 | 0.332 | 0.210 |  | 0.177 |
| **Ch19** | 0.189 | 0.209 | 0.314 | 0.134 | 0.273 | 0.229 | 0.379 | 0.224 | 0.360 | 0.290 | 0.296 | 0.208 | 0.288 | 0.094 | 0.143 | 0.017 | 0.491 | 0.177 |  |

**Table S2 Data corresponding to brain network connections of ID**

|  | **Ch1** | **Ch2** | **Ch3** | **Ch4** | **Ch5** | **Ch6** | **Ch7** | **Ch8** | **Ch9** | **Ch10** | **Ch11** | **Ch12** | **Ch13** | **Ch14** | **Ch15** | **Ch16** | **Ch17** | **Ch18** | **Ch19** |
| --- | --- | --- | --- | --- | --- | --- | --- | --- | --- | --- | --- | --- | --- | --- | --- | --- | --- | --- | --- |
| **Ch1** |  | 0.395 | 0.195 | 0.049 | 0.084 | 0.098 | 0.149 | 0.014 | 0.205 | 0.273 | 0.247 | 0.182 | 0.136 | -0.028 | 0.089 | 0.062 | 0.184 | 0.124 | 0.144 |
| **Ch2** | 0.395 |  | 0.112 | 0.126 | 0.058 | 0.183 | 0.122 | 0.131 | 0.376 | 0.353 | 0.181 | 0.188 | 0.095 | -0.048 | -0.115 | -0.035 | 0.385 | 0.270 | 0.246 |
| **Ch3** | 0.195 | 0.112 |  | 0.465 | 0.620 | 0.246 | 0.481 | 0.413 | 0.537 | 0.283 | 0.399 | -0.069 | 0.412 | 0.256 | 0.295 | 0.269 | 0.470 | 0.133 | 0.371 |
| **Ch4** | 0.049 | 0.126 | 0.465 |  | 0.415 | 0.382 | 0.397 | 0.218 | 0.278 | 0.174 | 0.403 | -0.204 | 0.241 | 0.254 | 0.096 | 0.204 | 0.364 | 0.165 | 0.244 |
| **Ch5** | 0.084 | 0.058 | 0.620 | 0.415 |  | 0.287 | 0.323 | 0.518 | 0.352 | 0.118 | 0.170 | 0.096 | 0.367 | 0.311 | 0.384 | 0.232 | 0.332 | 0.122 | 0.199 |
| **Ch6** | 0.098 | 0.183 | 0.246 | 0.382 | 0.287 |  | 0.356 | 0.304 | 0.163 | 0.152 | 0.247 | 0.039 | 0.179 | 0.307 | 0.229 | 0.298 | 0.177 | 0.184 | 0.322 |
| **Ch7** | 0.149 | 0.122 | 0.481 | 0.397 | 0.323 | 0.356 |  | 0.261 | 0.384 | 0.262 | 0.164 | -0.210 | 0.211 | 0.239 | 0.181 | 0.280 | 0.565 | 0.251 | 0.330 |
| **Ch8** | 0.014 | 0.131 | 0.413 | 0.218 | 0.518 | 0.304 | 0.261 |  | 0.464 | 0.018 | 0.117 | 0.094 | 0.395 | 0.356 | 0.338 | 0.257 | 0.347 | 0.272 | 0.272 |
| **Ch9** | 0.205 | 0.376 | 0.537 | 0.278 | 0.352 | 0.163 | 0.384 | 0.464 |  | 0.325 | 0.417 | 0.057 | 0.373 | 0.173 | 0.114 | 0.071 | 0.524 | 0.298 | 0.576 |
| **Ch10** | 0.273 | 0.353 | 0.283 | 0.174 | 0.118 | 0.152 | 0.262 | 0.018 | 0.325 |  | 0.272 | 0.108 | 0.182 | -0.030 | -0.089 | -0.003 | 0.274 | 0.169 | 0.310 |
| **Ch11** | 0.247 | 0.181 | 0.399 | 0.403 | 0.170 | 0.247 | 0.164 | 0.117 | 0.417 | 0.272 |  | 0.032 | 0.188 | 0.150 | 0.149 | 0.044 | 0.223 | 0.111 | 0.374 |
| **Ch12** | 0.182 | 0.188 | -0.069 | -0.204 | 0.096 | 0.039 | -0.210 | 0.094 | 0.057 | 0.108 | 0.032 |  | 0.081 | 0.037 | 0.061 | 0.096 | -0.035 | -0.059 | 0.046 |
| **Ch13** | 0.136 | 0.095 | 0.412 | 0.241 | 0.367 | 0.179 | 0.211 | 0.395 | 0.373 | 0.182 | 0.188 | 0.081 |  | 0.249 | 0.409 | 0.306 | 0.370 | 0.431 | 0.395 |
| **Ch14** | -0.028 | -0.048 | 0.256 | 0.254 | 0.311 | 0.307 | 0.239 | 0.356 | 0.173 | -0.030 | 0.150 | 0.037 | 0.249 |  | 0.419 | 0.459 | 0.215 | 0.348 | 0.298 |
| **Ch15** | 0.089 | -0.115 | 0.295 | 0.096 | 0.384 | 0.229 | 0.181 | 0.338 | 0.114 | -0.089 | 0.149 | 0.061 | 0.409 | 0.419 |  | 0.368 | 0.150 | 0.088 | 0.088 |
| **Ch16** | 0.062 | -0.035 | 0.269 | 0.204 | 0.232 | 0.298 | 0.280 | 0.257 | 0.071 | -0.003 | 0.044 | 0.096 | 0.306 | 0.459 | 0.368 |  | 0.200 | 0.340 | 0.154 |
| **Ch17** | 0.184 | 0.385 | 0.470 | 0.364 | 0.332 | 0.177 | 0.565 | 0.347 | 0.524 | 0.274 | 0.223 | -0.035 | 0.370 | 0.215 | 0.150 | 0.200 |  | 0.457 | 0.531 |
| **Ch18** | 0.124 | 0.270 | 0.133 | 0.165 | 0.122 | 0.184 | 0.251 | 0.272 | 0.298 | 0.169 | 0.111 | -0.059 | 0.431 | 0.348 | 0.088 | 0.340 | 0.457 |  | 0.404 |
| **Ch19** | 0.144 | 0.246 | 0.371 | 0.244 | 0.199 | 0.322 | 0.330 | 0.272 | 0.576 | 0.310 | 0.374 | 0.046 | 0.395 | 0.298 | 0.088 | 0.154 | 0.531 | 0.404 |  |

**Table S3 Group-level statistics for all 171 functional connectivity (channel-pairwise) comparisons.**

| **Channel to channel** | **Connectivity strength (M ± SD)** | | | **t-value** | **p (uncorrected)** | **p (FDR-corrected)** | **Significant (q < 0.05)** |
| --- | --- | --- | --- | --- | --- | --- | --- |
|  | **ASD group** | **ID group** | |  |  |  |  |
| 1~2 | 0.31 ± 0.57 | | 0.40 ± 0.49 | -0.5718 | 0.5699 | 0.9849 | No |
| 1~3 | 0.01 ± 0.57 | | 0.20 ± 0.51 | -1.215 | 0.2299 | 0.8569 | No |
| 1~4 | 0.02 ± 0.52 | | 0.05 ± 0.54 | -0.1659 | 0.8689 | 0.9862 | No |
| 1~5 | 0.17 ± 0.58 | | 0.08 ± 0.57 | 0.535 | 0.5949 | 0.9849 | No |
| 1~6 | 0.10 ± 0.54 | | 0.10 ± 0.58 | 0.0156 | 0.9876 | 0.9904 | No |
| 1~7 | 0.27 ± 0.47 | | 0.15 ± 0.58 | 0.8322 | 0.4091 | 0.97 | No |
| 1~8 | -0.02 ± 0.47 | | 0.01 ± 0.63 | -0.2372 | 0.814 | 0.9862 | No |
| 1~9 | 0.06 ± 0.49 | | 0.20 ± 0.54 | -0.9925 | 0.3255 | 0.9185 | No |
| 1~10 | 0.08 ± 0.58 | | 0.27 ± 0.48 | -1.2604 | 0.2131 | 0.8532 | No |
| 1~11 | 0.12 ± 0.56 | | 0.25 ± 0.53 | -0.8485 | 0.4001 | 0.97 | No |
| 1~12 | 0.10 ± 0.55 | | 0.18 ± 0.58 | -0.5504 | 0.5844 | 0.9849 | No |
| 1~13 | 0.10 ± 0.56 | | 0.14 ± 0.56 | -0.258 | 0.7974 | 0.9862 | No |
| 1~14 | -0.04 ± 0.53 | | -0.03 ± 0.56 | -0.0692 | 0.9451 | 0.9862 | No |
| 1~15 | 0.05 ± 0.52 | | 0.09 ± 0.65 | -0.2232 | 0.8246 | 0.9862 | No |
| 1~16 | 0.10 ± 0.55 | | 0.06 ± 0.68 | 0.1975 | 0.8442 | 0.9862 | No |
| 1~17 | 0.10 ± 0.49 | | 0.18 ± 0.51 | -0.5926 | 0.556 | 0.9849 | No |
| 1~18 | 0.14 ± 0.54 | | 0.12 ± 0.56 | 0.1017 | 0.9194 | 0.9862 | No |
| 1~19 | 0.19 ± 0.49 | | 0.14 ± 0.46 | 0.3378 | 0.7369 | 0.9862 | No |
| 2~3 | 0.06 ± 0.56 | | 0.11 ± 0.44 | -0.3923 | 0.6965 | 0.9862 | No |
| 2~4 | 0.22 ± 0.46 | | 0.13 ± 0.56 | 0.6913 | 0.4924 | 0.9849 | No |
| 2~5 | 0.06 ± 0.54 | | 0.06 ± 0.51 | 0.0121 | 0.9904 | 0.9904 | No |
| 2~6 | 0.10 ± 0.56 | | 0.18 ± 0.52 | -0.5221 | 0.6038 | 0.9849 | No |
| 2~7 | 0.15 ± 0.50 | | 0.12 ± 0.53 | 0.2003 | 0.842 | 0.9862 | No |
| 2~8 | 0.03 ± 0.52 | | 0.13 ± 0.56 | -0.7023 | 0.4856 | 0.9849 | No |
| 2~9 | -0.06 ± 0.48 | | 0.38 ± 0.47 | -3.3085 | 0.0017 | 0.0973 | No |
| 2~10 | 0.06 ± 0.53 | | 0.35 ± 0.32 | -2.5127 | 0.0151 | 0.3695 | No |
| 2~11 | -0.02 ± 0.53 | | 0.18 ± 0.51 | -1.3534 | 0.1818 | 0.797 | No |
| 2~12 | 0.24 ± 0.49 | | 0.19 ± 0.52 | 0.3535 | 0.7252 | 0.9862 | No |
| 2~13 | 0.19 ± 0.52 | | 0.09 ± 0.47 | 0.6783 | 0.5006 | 0.9849 | No |
| 2~14 | 0.03 ± 0.59 | | -0.05 ± 0.50 | 0.5143 | 0.6092 | 0.9849 | No |
| 2~15 | -0.03 ± 0.56 | | -0.11 ± 0.57 | 0.5563 | 0.5804 | 0.9849 | No |
| 2~16 | 0.02 ± 0.57 | | -0.03 ± 0.60 | 0.3301 | 0.7427 | 0.9862 | No |
| 2~17 | 0.00 ± 0.55 | | 0.38 ± 0.38 | -3.0645 | 0.0035 | 0.1184 | No |
| 2~18 | -0.05 ± 0.55 | | 0.27 ± 0.51 | -2.1417 | 0.0369 | 0.5862 | No |
| 2~19 | 0.21 ± 0.48 | | 0.25 ± 0.49 | -0.2701 | 0.7882 | 0.9862 | No |
| 3~4 | 0.34 ± 0.39 | | 0.46 ± 0.36 | -1.1502 | 0.2553 | 0.8732 | No |
| 3~5 | 0.28 ± 0.61 | | 0.62 ± 0.29 | -2.7606 | 0.0081 | 0.2306 | No |
| 3~6 | 0.27 ± 0.51 | | 0.25 ± 0.44 | 0.1548 | 0.8776 | 0.9862 | No |
| 3~7 | 0.33 ± 0.54 | | 0.48 ± 0.34 | -1.2428 | 0.2195 | 0.8532 | No |
| 3~8 | 0.21 ± 0.56 | | 0.41 ± 0.49 | -1.3345 | 0.1878 | 0.8031 | No |
| 3~9 | 0.27 ± 0.48 | | 0.54 ± 0.37 | -2.1989 | 0.0324 | 0.5862 | No |
| 3~10 | 0.22 ± 0.51 | | 0.28 ± 0.56 | -0.4243 | 0.6731 | 0.9862 | No |
| 3~11 | 0.37 ± 0.58 | | 0.40 ± 0.50 | -0.1671 | 0.8679 | 0.9862 | No |
| 3~12 | 0.21 ± 0.56 | | -0.07 ± 0.55 | 1.7816 | 0.0807 | 0.6876 | No |
| 3~13 | 0.43 ± 0.52 | | 0.41 ± 0.45 | 0.1492 | 0.882 | 0.9862 | No |
| 3~14 | 0.11 ± 0.55 | | 0.26 ± 0.47 | -1.0192 | 0.3128 | 0.9185 | No |
| 3~15 | 0.15 ± 0.56 | | 0.29 ± 0.51 | -0.956 | 0.3435 | 0.9185 | No |
| 3~16 | -0.02 ± 0.50 | | 0.27 ± 0.49 | -2.0857 | 0.0419 | 0.5975 | No |
| 3~17 | 0.26 ± 0.54 | | 0.47 ± 0.42 | -1.5311 | 0.1318 | 0.7043 | No |
| 3~18 | 0.14 ± 0.57 | | 0.13 ± 0.45 | 0.0806 | 0.9361 | 0.9862 | No |
| 3~19 | 0.31 ± 0.46 | | 0.37 ± 0.52 | -0.4294 | 0.6694 | 0.9862 | No |
| 4~5 | 0.24 ± 0.60 | | 0.42 ± 0.42 | -1.1675 | 0.2483 | 0.8703 | No |
| 4~6 | 0.15 ± 0.57 | | 0.38 ± 0.44 | -1.6225 | 0.1108 | 0.6912 | No |
| 4~7 | 0.42 ± 0.53 | | 0.40 ± 0.60 | 0.1496 | 0.8817 | 0.9862 | No |
| 4~8 | 0.27 ± 0.48 | | 0.22 ± 0.66 | 0.2865 | 0.7763 | 0.9862 | No |
| 4~9 | 0.22 ± 0.46 | | 0.28 ± 0.53 | -0.4023 | 0.6891 | 0.9862 | No |
| 4~10 | 0.08 ± 0.53 | | 0.17 ± 0.48 | -0.6555 | 0.515 | 0.9849 | No |
| 4~11 | 0.16 ± 0.44 | | 0.40 ± 0.49 | -1.8896 | 0.0644 | 0.6876 | No |
| 4~12 | 0.34 ± 0.46 | | -0.20 ± 0.51 | 4.0781 | 0.0002 | 0.0134 | Yes |
| 4~13 | 0.25 ± 0.46 | | 0.24 ± 0.52 | 0.1054 | 0.9165 | 0.9862 | No |
| 4~14 | 0.25 ± 0.56 | | 0.25 ± 0.60 | -0.0258 | 0.9795 | 0.9904 | No |
| 4~15 | 0.08 ± 0.52 | | 0.10 ± 0.62 | -0.0996 | 0.921 | 0.9862 | No |
| 4~16 | 0.09 ± 0.54 | | 0.20 ± 0.60 | -0.7301 | 0.4686 | 0.9849 | No |
| 4~17 | 0.39 ± 0.53 | | 0.36 ± 0.50 | 0.1776 | 0.8597 | 0.9862 | No |
| 4~18 | 0.10 ± 0.55 | | 0.17 ± 0.67 | -0.4034 | 0.6883 | 0.9862 | No |
| 4~19 | 0.13 ± 0.44 | | 0.24 ± 0.51 | -0.8369 | 0.4065 | 0.97 | No |
| 5~6 | 0.29 ± 0.54 | | 0.29 ± 0.50 | 0.02 | 0.9842 | 0.9904 | No |
| 5~7 | 0.52 ± 0.47 | | 0.32 ± 0.53 | 1.4163 | 0.1626 | 0.7564 | No |
| 5~8 | 0.24 ± 0.51 | | 0.52 ± 0.38 | -2.1325 | 0.0377 | 0.5862 | No |
| 5~9 | 0.13 ± 0.54 | | 0.35 ± 0.47 | -1.5419 | 0.1292 | 0.7043 | No |
| 5~10 | 0.01 ± 0.61 | | 0.12 ± 0.57 | -0.6252 | 0.5346 | 0.9849 | No |
| 5~11 | 0.23 ± 0.59 | | 0.17 ± 0.53 | 0.3634 | 0.7178 | 0.9862 | No |
| 5~12 | 0.21 ± 0.58 | | 0.10 ± 0.50 | 0.7144 | 0.4781 | 0.9849 | No |
| 5~13 | 0.44 ± 0.47 | | 0.37 ± 0.46 | 0.5976 | 0.5527 | 0.9849 | No |
| 5~14 | 0.32 ± 0.58 | | 0.31 ± 0.46 | 0.0578 | 0.9542 | 0.9862 | No |
| 5~15 | 0.48 ± 0.45 | | 0.38 ± 0.58 | 0.6553 | 0.5152 | 0.9849 | No |
| 5~16 | 0.22 ± 0.53 | | 0.23 ± 0.54 | -0.1053 | 0.9166 | 0.9862 | No |
| 5~17 | 0.30 ± 0.61 | | 0.33 ± 0.54 | -0.1888 | 0.851 | 0.9862 | No |
| 5~18 | 0.41 ± 0.49 | | 0.12 ± 0.59 | 1.9704 | 0.0541 | 0.6876 | No |
| 5~19 | 0.27 ± 0.47 | | 0.20 ± 0.54 | 0.5377 | 0.5931 | 0.9849 | No |
| 6~7 | 0.24 ± 0.54 | | 0.36 ± 0.53 | -0.7894 | 0.4335 | 0.9849 | No |
| 6~8 | 0.45 ± 0.37 | | 0.30 ± 0.54 | 1.0685 | 0.2933 | 0.9119 | No |
| 6~9 | 0.17 ± 0.46 | | 0.16 ± 0.59 | 0.0465 | 0.9631 | 0.9862 | No |
| 6~10 | 0.17 ± 0.50 | | 0.15 ± 0.52 | 0.1217 | 0.9036 | 0.9862 | No |
| 6~11 | 0.19 ± 0.49 | | 0.25 ± 0.52 | -0.4414 | 0.6607 | 0.9862 | No |
| 6~12 | 0.05 ± 0.57 | | 0.04 ± 0.44 | 0.0839 | 0.9335 | 0.9862 | No |
| 6~13 | 0.28 ± 0.46 | | 0.18 ± 0.44 | 0.8233 | 0.4141 | 0.97 | No |
| 6~14 | 0.12 ± 0.66 | | 0.31 ± 0.51 | -1.0902 | 0.2807 | 0.9051 | No |
| 6~15 | 0.10 ± 0.61 | | 0.23 ± 0.57 | -0.766 | 0.4472 | 0.9849 | No |
| 6~16 | -0.18 ± 0.54 | | 0.30 ± 0.55 | -3.1712 | 0.0025 | 0.1088 | No |
| 6~17 | 0.21 ± 0.53 | | 0.18 ± 0.57 | 0.2193 | 0.8273 | 0.9862 | No |
| 6~18 | 0.07 ± 0.59 | | 0.18 ± 0.58 | -0.6719 | 0.5046 | 0.9849 | No |
| 6~19 | 0.23 ± 0.47 | | 0.32 ± 0.53 | -0.6772 | 0.5013 | 0.9849 | No |
| 7~8 | 0.28 ± 0.56 | | 0.26 ± 0.63 | 0.0864 | 0.9315 | 0.9862 | No |
| 7~9 | 0.22 ± 0.49 | | 0.38 ± 0.52 | -1.1648 | 0.2494 | 0.8703 | No |
| 7~10 | 0.08 ± 0.52 | | 0.26 ± 0.48 | -1.3196 | 0.1927 | 0.8039 | No |
| 7~11 | 0.23 ± 0.52 | | 0.16 ± 0.51 | 0.4624 | 0.6457 | 0.9862 | No |
| 7~12 | 0.32 ± 0.46 | | -0.21 ± 0.44 | 4.1945 | 0.0001 | 0.0134 | Yes |
| 7~13 | 0.35 ± 0.40 | | 0.21 ± 0.49 | 1.119 | 0.2683 | 0.8995 | No |
| 7~14 | 0.19 ± 0.57 | | 0.24 ± 0.52 | -0.3116 | 0.7566 | 0.9862 | No |
| 7~15 | 0.28 ± 0.51 | | 0.18 ± 0.58 | 0.6655 | 0.5087 | 0.9849 | No |
| 7~16 | 0.04 ± 0.52 | | 0.28 ± 0.56 | -1.6231 | 0.1106 | 0.6912 | No |
| 7~17 | 0.50 ± 0.48 | | 0.56 ± 0.47 | -0.5135 | 0.6098 | 0.9849 | No |
| 7~18 | 0.26 ± 0.53 | | 0.25 ± 0.62 | 0.0771 | 0.9388 | 0.9862 | No |
| 7~19 | 0.38 ± 0.39 | | 0.33 ± 0.52 | 0.3968 | 0.6931 | 0.9862 | No |
| 8~9 | 0.28 ± 0.40 | | 0.46 ± 0.43 | -1.5938 | 0.117 | 0.6912 | No |
| 8~10 | 0.10 ± 0.44 | | 0.02 ± 0.66 | 0.4905 | 0.6273 | 0.9862 | No |
| 8~11 | 0.30 ± 0.48 | | 0.12 ± 0.58 | 1.256 | 0.2147 | 0.8532 | No |
| 8~12 | 0.24 ± 0.51 | | 0.09 ± 0.55 | 1.0157 | 0.3145 | 0.9185 | No |
| 8~13 | 0.18 ± 0.50 | | 0.39 ± 0.43 | -1.593 | 0.1172 | 0.6912 | No |
| 8~14 | 0.26 ± 0.52 | | 0.36 ± 0.57 | -0.6146 | 0.5415 | 0.9849 | No |
| 8~15 | 0.11 ± 0.57 | | 0.34 ± 0.58 | -1.4336 | 0.1577 | 0.7564 | No |
| 8~16 | 0.02 ± 0.50 | | 0.26 ± 0.65 | -1.4232 | 0.1637 | 0.7564 | No |
| 8~17 | 0.32 ± 0.52 | | 0.35 ± 0.56 | -0.1928 | 0.8479 | 0.9862 | No |
| 8~18 | 0.16 ± 0.58 | | 0.27 ± 0.67 | -0.6556 | 0.515 | 0.9849 | No |
| 8~19 | 0.22 ± 0.46 | | 0.27 ± 0.56 | -0.3421 | 0.7337 | 0.9862 | No |
| 9~10 | 0.14 ± 0.51 | | 0.33 ± 0.64 | -1.1721 | 0.2465 | 0.8703 | No |
| 9~11 | 0.26 ± 0.53 | | 0.42 ± 0.51 | -1.0784 | 0.2858 | 0.9051 | No |
| 9~12 | 0.25 ± 0.46 | | 0.06 ± 0.58 | 1.3775 | 0.1742 | 0.7841 | No |
| 9~13 | 0.24 ± 0.45 | | 0.37 ± 0.41 | -1.0931 | 0.2794 | 0.9051 | No |
| 9~14 | 0.12 ± 0.54 | | 0.17 ± 0.57 | -0.3684 | 0.714 | 0.9862 | No |
| 9~15 | 0.03 ± 0.48 | | 0.11 ± 0.55 | -0.5591 | 0.5785 | 0.9849 | No |
| 9~16 | 0.15 ± 0.45 | | 0.07 ± 0.60 | 0.5191 | 0.607 | 0.9849 | No |
| 9~17 | 0.41 ± 0.45 | | 0.52 ± 0.41 | -0.9342 | 0.3545 | 0.9185 | No |
| 9~18 | 0.27 ± 0.50 | | 0.30 ± 0.51 | -0.1965 | 0.845 | 0.9862 | No |
| 9~19 | 0.36 ± 0.42 | | 0.58 ± 0.41 | -1.858 | 0.0688 | 0.6876 | No |
| 10~11 | 0.25 ± 0.51 | | 0.27 ± 0.60 | -0.165 | 0.8695 | 0.9862 | No |
| 10~12 | 0.02 ± 0.52 | | 0.11 ± 0.63 | -0.5772 | 0.5663 | 0.9849 | No |
| 10~13 | 0.09 ± 0.51 | | 0.18 ± 0.58 | -0.587 | 0.5598 | 0.9849 | No |
| 10~14 | 0.00 ± 0.56 | | -0.03 ± 0.55 | 0.2012 | 0.8414 | 0.9862 | No |
| 10~15 | 0.13 ± 0.52 | | -0.09 ± 0.55 | 1.4931 | 0.1414 | 0.7329 | No |
| 10~16 | -0.01 ± 0.53 | | 0.00 ± 0.59 | -0.0559 | 0.9557 | 0.9862 | No |
| 10~17 | 0.03 ± 0.54 | | 0.27 ± 0.45 | -1.7473 | 0.0865 | 0.6876 | No |
| 10~18 | -0.05 ± 0.56 | | 0.17 ± 0.51 | -1.4579 | 0.1509 | 0.7564 | No |
| 10~19 | 0.29 ± 0.45 | | 0.31 ± 0.57 | -0.1479 | 0.883 | 0.9862 | No |
| 11~12 | 0.30 ± 0.59 | | 0.03 ± 0.59 | 1.6105 | 0.1133 | 0.6912 | No |
| 11~13 | 0.34 ± 0.54 | | 0.19 ± 0.56 | 1.024 | 0.3106 | 0.9185 | No |
| 11~14 | 0.14 ± 0.57 | | 0.15 ± 0.58 | -0.0493 | 0.9609 | 0.9862 | No |
| 11~15 | 0.29 ± 0.48 | | 0.15 ± 0.63 | 0.957 | 0.343 | 0.9185 | No |
| 11~16 | 0.12 ± 0.51 | | 0.04 ± 0.61 | 0.4823 | 0.6316 | 0.9862 | No |
| 11~17 | 0.28 ± 0.55 | | 0.22 ± 0.56 | 0.3855 | 0.7015 | 0.9862 | No |
| 11~18 | 0.21 ± 0.52 | | 0.11 ± 0.57 | 0.6689 | 0.5065 | 0.9849 | No |
| 11~19 | 0.30 ± 0.48 | | 0.37 ± 0.47 | -0.5879 | 0.5591 | 0.9849 | No |
| 12~13 | 0.33 ± 0.56 | | 0.08 ± 0.42 | 1.7303 | 0.0895 | 0.6876 | No |
| 12~14 | 0.31 ± 0.50 | | 0.04 ± 0.55 | 1.8595 | 0.0686 | 0.6876 | No |
| 12~15 | 0.10 ± 0.57 | | 0.06 ± 0.56 | 0.2181 | 0.8282 | 0.9862 | No |
| 12~16 | 0.11 ± 0.50 | | 0.10 ± 0.56 | 0.1286 | 0.8982 | 0.9862 | No |
| 12~17 | 0.24 ± 0.53 | | -0.04 ± 0.46 | 1.9516 | 0.0564 | 0.6876 | No |
| 12~18 | 0.27 ± 0.54 | | -0.06 ± 0.48 | 2.2578 | 0.0282 | 0.5862 | No |
| 12~19 | 0.21 ± 0.46 | | 0.05 ± 0.51 | 1.2133 | 0.2305 | 0.8569 | No |
| 13~14 | 0.18 ± 0.54 | | 0.25 ± 0.51 | -0.4625 | 0.6456 | 0.9862 | No |
| 13~15 | 0.38 ± 0.46 | | 0.41 ± 0.45 | -0.2179 | 0.8283 | 0.9862 | No |
| 13~16 | 0.23 ± 0.51 | | 0.31 ± 0.57 | -0.5125 | 0.6105 | 0.9849 | No |
| 13~17 | 0.24 ± 0.54 | | 0.37 ± 0.46 | -0.9344 | 0.3544 | 0.9185 | No |
| 13~18 | 0.20 ± 0.49 | | 0.43 ± 0.44 | -1.714 | 0.0925 | 0.6876 | No |
| 13~19 | 0.29 ± 0.45 | | 0.40 ± 0.43 | -0.865 | 0.391 | 0.969 | No |
| 14~15 | 0.27 ± 0.58 | | 0.42 ± 0.50 | -0.9676 | 0.3377 | 0.9185 | No |
| 14~16 | 0.34 ± 0.52 | | 0.46 ± 0.36 | -0.8866 | 0.3794 | 0.9619 | No |
| 14~17 | 0.08 ± 0.59 | | 0.22 ± 0.52 | -0.8808 | 0.3825 | 0.9619 | No |
| 14~18 | 0.31 ± 0.57 | | 0.35 ± 0.51 | -0.2543 | 0.8003 | 0.9862 | No |
| 14~19 | 0.09 ± 0.46 | | 0.30 ± 0.50 | -1.533 | 0.1313 | 0.7043 | No |
| 15~16 | 0.29 ± 0.52 | | 0.37 ± 0.58 | -0.5164 | 0.6078 | 0.9849 | No |
| 15~17 | 0.07 ± 0.54 | | 0.15 ± 0.60 | -0.4879 | 0.6277 | 0.9862 | No |
| 15~18 | 0.25 ± 0.54 | | 0.09 ± 0.65 | 0.9814 | 0.3309 | 0.9185 | No |
| 15~19 | 0.14 ± 0.45 | | 0.09 ± 0.55 | 0.4063 | 0.6862 | 0.9862 | No |
| 16~17 | -0.05 ± 0.52 | | 0.20 ± 0.51 | -1.7504 | 0.0859 | 0.6876 | No |
| 16~18 | 0.33 ± 0.50 | | 0.34 ± 0.62 | -0.0511 | 0.9594 | 0.9862 | No |
| 16~19 | 0.02 ± 0.48 | | 0.15 ± 0.57 | -0.9528 | 0.3451 | 0.9185 | No |
| 17~18 | 0.21 ± 0.57 | | 0.46 ± 0.48 | -1.6469 | 0.1056 | 0.6912 | No |
| 17~19 | 0.49 ± 0.41 | | 0.53 ± 0.46 | -0.3344 | 0.7394 | 0.9862 | No |
| 18~19 | 0.18 ± 0.48 | | 0.40 ± 0.41 | -1.7844 | 0.0802 | 0.6876 | No |

**Table S4 Associations between channel Oxy-Hb changes and demographic characteristics in the ASD group.**

| Channel names | Age | | Being an  only child  (Yes/No) | | Parental care  (Yes/No) | | Gender (Male/Female) | | Natural birth (yes/no) | |
| --- | --- | --- | --- | --- | --- | --- | --- | --- | --- | --- |
|  | *r* | *p* | *r_s_* | *p* | *r_s_* | *p* | *r_s_* | *p* | *r_s_* | *p* |
| Ch1 | -0.175 | 0.331 | -0.077 | 0.669 | -0.050 | 0.782 | 0.011 | 0.951 | -0.033 | 0.855 |
| Ch2 | -0.079 | 0.661 | -0.084 | 0.643 | 0.021 | 0.906 | -0.033 | 0.854 | -0.073 | 0.687 |
| Ch3 | -0.144 | 0.426 | 0.000 | 1.000 | 0.021 | 0.906 | -0.089 | 0.624 | -0.099 | 0.583 |
| Ch4 | -0.245 | 0.170 | -0.258 | 0.148 | -0.179 | 0.320 | -0.255 | 0.153 | 0.000 | 1.000 |
| Ch5 | 0.125 | 0.490 | 0.071 | 0.695 | 0.114 | 0.526 | 0.055 | 0.760 | -0.040 | 0.826 |
| Ch6 | -0.155 | 0.390 | -0.097 | 0.593 | -0.286 | 0.107 | 0.011 | 0.951 | -0.106 | 0.558 |
| Ch7 | -0.379 | 0.030 | -0.071 | 0.695 | 0.014 | 0.937 | -0.199 | 0.266 | -0.337 | 0.055 |
| Ch8 | -0.325 | 0.065 | -0.174 | 0.333 | -0.107 | 0.553 | -0.089 | 0.624 | -0.430 | 0.012 |
| Ch9 | -0.209 | 0.244 | 0.122 | 0.498 | 0.422 | 0.015 | -0.089 | 0.624 | -0.245 | 0.170 |
| Ch10 | -0.264 | 0.138 | -0.219 | 0.221 | 0.036 | 0.844 | -0.122 | 0.500 | -0.265 | 0.173 |
| Ch11 | -0.239 | 0.181 | -0.058 | 0.749 | 0.000 | 1.000 | 0.199 | 0.266 | -0.238 | 0.182 |
| Ch12 | -0.188 | 0.296 | -0.116 | 0.521 | 0.157 | 0.382 | -0.232 | 0.193 | 0.046 | 0.798 |
| Ch13 | -0.412 | 0.017 | -0.052 | 0.776 | 0.379 | 0.030 | -0.310 | 0.079 | 0.079 | 0.661 |
| Ch14 | -0.381 | 0.029 | 0.161 | 0.371 | -0.093 | 0.607 | 0.022 | 0.903 | 0.093 | 0.608 |
| Ch15 | -0.155 | 0.390 | -0.019 | 0.915 | 0.114 | 0.526 | 0.022 | 0.903 | 0.093 | 0.608 |
| Ch16 | -0.126 | 0.484 | -0.444 | 0.016 | -0.107 | 0.553 | -0.255 | 0.153 | 0.013 | 0.942 |
| Ch17 | -0.258 | 0.147 | 0.008 | 0.966 | 0.043 | 0.813 | -0.155 | 0.389 | -0.278 | 0.117 |
| Ch18 | -0.110 | 0.543 | 0.059 | 0.763 | 0.100 | 0.580 | -0.244 | 0.172 | -0.331 | 0.060 |
| Ch19 | -0.293 | 0.098 | -0.192 | 0.317 | 0.186 | 0.301 | -0.100 | 0.581 | -0351 | 0.045 |

**Table S5** **Associations between channel Oxy-Hb changes and clinical characteristics in the ASD group.**

| Channel | Full-Scale Intelligence Quotient (FSIQ) | | Childhood Autism Rating Scale (CARS) | | Correct Words / Minute | | Perseverations (count) / Minute | |
| --- | --- | --- | --- | --- | --- | --- | --- | --- |
|  | *r* | *p* | *r* | *p* | *r_s_* | *p* | *r_s_* | *p* |
| 1 | 0.061 | 0.735 | -0.852 | **<0.001** | 0.032 | 0.860 | 0.064 | 0.722 |
| 2 | -0.180 | 0.316 | -0.606 | **<0.001** | 0.128 | 0.478 | -0.058 | 0.749 |
| 3 | -0.096 | 0.595 | 0.198 | 0.270 | 0.153 | 0.394 | -0.225 | 0.207 |
| 4 | -0.030 | 0.869 | 0.115 | 0.525 | 0.192 | 0.285 | -0.109 | 0.554 |
| 5 | -0.221 | 0.217 | -0.282 | 0.111 | 0.153 | 0.394 | -0.238 | 0.182 |
| 6 | 0.043 | 0.811 | 0.072 | 0.690 | 0.371 | **0.034** | -0.090 | 0.618 |
| 7 | -0.090 | 0.619 | 0.122 | 0.499 | 0.403 | **0.020** | -0.109 | 0.544 |
| 8 | -0.189 | 0.291 | 0.043 | 0.811 | 0.320 | 0.070 | -0.322 | 0.068 |
| 9 | -0.212 | 0.236 | 0.113 | 0.531 | 0.320 | 0.070 | -0.161 | 0.371 |
| 10 | -0.201 | 0.263 | -0.067 | 0.713 | 0.141 | 0.435 | 0.064 | 0.722 |
| 11 | -0.114 | 0.529 | -0.012 | 0.945 | 0.173 | 0.337 | 0.032 | 0.859 |
| 12 | 0.215 | 0.230 | -0.186 | 0.301 | 0.083 | 0.646 | 0.174 | 0.333 |
| 13 | 0.025 | 0.890 | -0.453 | **0.008** | -0.102 | 0.571 | 0.303 | 0.087 |
| 14 | 0.181 | 0.312 | -0.311 | 0.078 | 0.345 | **0.049** | -0.200 | 0.265 |
| 15 | -0.082 | 0.649 | -0.180 | 0.317 | 0.051 | 0.777 | -0.006 | 0.654 |
| 16 | -0.118 | 0.512 | 0.222 | 0.215 | 0.396 | **0.022** | 0.019 | 0.915 |
| 17 | 0.002 | 0.992 | 0.014 | 0.939 | 0.173 | 0.337 | -0.187 | 0.298 |
| 18 | -0.281 | 0.113 | 0.356 | **0.042** | 0.256 | 0.151 | 0.052 | 0.776 |
| 19 | -0.134 | 0.458 | 0.085 | 0.640 | 0.230 | 0.198 | 0.090 | 0.618 |

**Table S6 MNI coordinates and anatomical labels for each fNIRS channel.**

| **Channel** | **Source–Detector** | **MNI-X** | **MNI-Y** | **MNI-Z** | **Brodmann area** | **Anatomical region (AAL)** |
| --- | --- | --- | --- | --- | --- | --- |
| 1 | S2–D2 | 52 | 22 | 12 | 44/45 | Right inferior frontal gyrus |
| 2 | S2–D7 | 48 | 26 | 8 | 44/45 | Right inferior frontal gyrus |
| 3 | S3–D2 | 42 | 46 | 16 | 10 | Right frontopolar cortex |
| 4 | S3–D3 | 36 | 52 | 18 | 10 | Right frontopolar cortex |
| 5 | S3–D8 | 30 | 58 | 14 | 10 | Right frontopolar cortex |
| 6 | S4–D3 | –24 | 62 | 10 | 10 | Left frontopolar cortex |
| 7 | S4–D4 | –18 | 64 | 12 | 10 | Left frontopolar cortex |
| 8 | S4–D9 | –12 | 60 | 16 | 10 | Left frontopolar cortex |
| 9 | S5–D4 | –8 | 56 | 22 | 44/45 | Left inferior frontal gyrus |
| 10 | S5–D10 | –6 | 54 | 24 | 45 | Left pars triangularis Broca's cortex/ Inferior prefrontal gyrus |
| 11 | S8–D2 | 14 | 58 | 18 | 10 | Right frontopolar cortex |
| 12 | S8–D7 | 22 | 60 | 12 | 9/46 | Right dorsolateral prefrontal cortex |
| 13 | S8–D8 | 30 | 56 | 10 | 10 | Right frontopolar cortex |
| 14 | S9–D3 | 38 | 52 | 14 | 10 | Right frontopolar cortex |
| 15 | S9–D8 | 44 | 48 | 16 | 10 | Right frontopolar cortex |
| 16 | S9–D9 | –50 | 42 | 18 | 10 | Left frontopolar cortex |
| 17 | S10–D4 | –54 | 34 | 12 | 10 | Left frontopolar cortex |
| 18 | S10–D9 | –52 | 26 | 8 | 10 | Left frontopolar cortex |
| 19 | S10–D10 | –48 | 38 | 18 | 9/46 | Left dorsolateral prefrontal cortex |
